# Supplementary material for: How climate policy commitments influence energy systems and the economies of US states
Source: Nat Commun. 2023 Aug 10;14:4850. doi: 10.1038/s41467-023-40560-y (PMC10415253; doi:10.1038/s41467-023-40560-y)
Supplement: Supplementary file 1 — Supplementary Information [file 41467_2023_40560_MOESM1_ESM.pdf]

Supplementary Information for: How climate policy commitments influence energy systems and the economy of US States

Parrish Bergquist* Christopher Warshaw†

June 29, 2023

Contents

1 Descriptive results **S-1**

2 Discrimination parameter estimates **S-4**

3 Validating the estimates of climate policy **S-6**

3.1 Convergent Validation S-6

3.2 Construct Validation S-6

4 Regression results **S-10**

5 Robustness checks **S-10**

6 Policy coding and data sources **S-14**

7 Dependent variables and covariates: Coding and data sources **S-17**

*Assistant Professor, Department of Political Science, University of Pennsylvania

†Associate Professor, Department of Political Science, George Washington University

1 Descriptive results

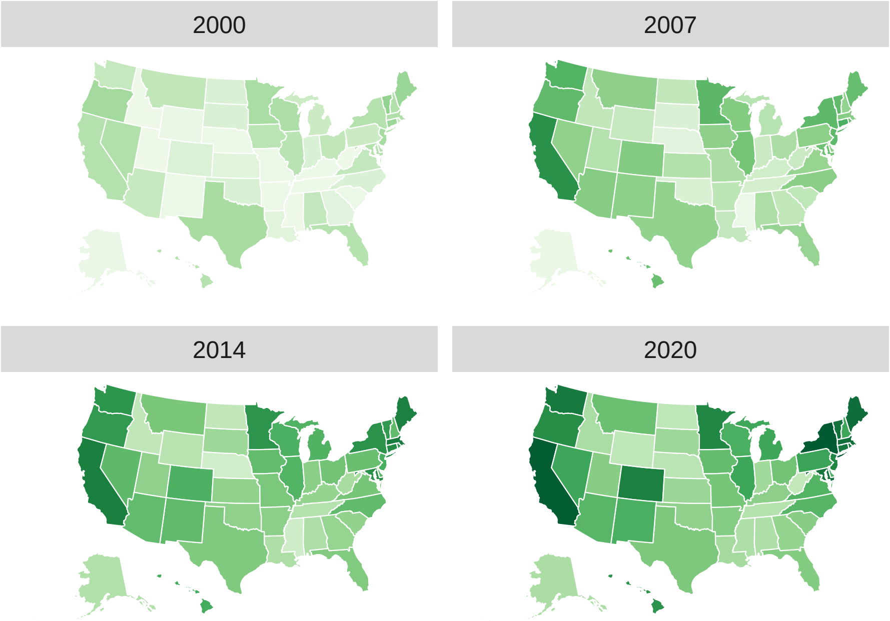

Figure S1: The figure complements Figure 1 in the main text by including Alaska and Hawaii.

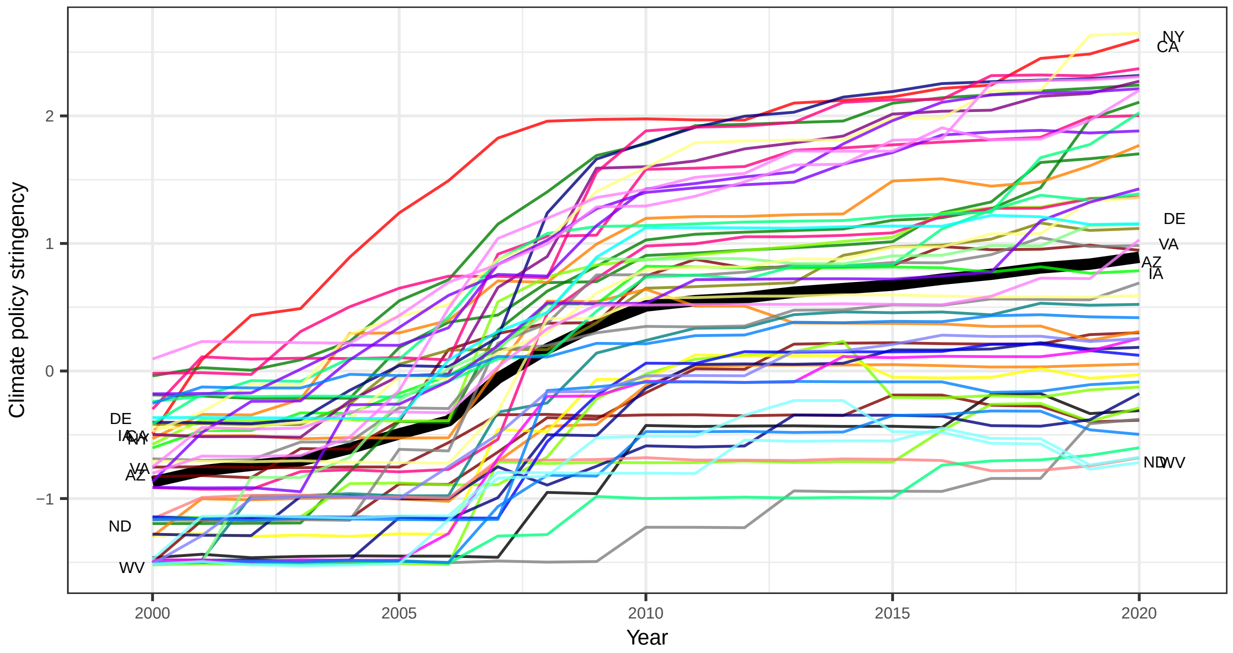

Figure S2: Increasing state climate policy commitments over time: Each thin line represents the trajectory of an individual state. The bold line shows the average of our climate policy stringency index across all states, with each state weighted equally. To illustrate change over time, we include labels for a selected group of states whose climate policy stringency spans the distribution in 2020.

Table S1 shows cross-sectional estimates of state climate policy stringency in 2000, 2010, and 2020, along with each state's ranking relative to other states in the same year.

State	Climate policy			Climate policy rank		
	2000	2010	2020	2000	2010	2020
AK	-1.46	-0.43	-0.31	39	43	44
AL	-0.75	-0.34	-0.39	24	42	46
AR	-1.50	-0.17	0.30	44	41	33
AZ	-0.83	0.74	0.95	26	23	26
CA	-0.49	1.98	2.60	15	1	2
CO	-1.20	1.03	2.11	35	15	10
CT	-0.04	1.78	2.24	3	4	7
DC	-0.19	0.91	1.70	5	17	15
DE	-0.42	0.65	1.12	12	25	23
FL	-0.50	0.64	0.31	17	26	32
GA	-1.30	-0.12	0.05	38	39	38
HI	-0.53	1.20	1.77	18	12	14
IA	-0.60	0.82	0.79	20	20	27
ID	-1.52	-0.72	-0.29	51	48	43
IL	-0.58	0.87	1.37	19	19	18
IN	-1.14	-0.02	-0.13	30	34	41
KS	-1.29	-0.06	-0.03	37	36	39
KY	-1.50	-0.14	0.19	46	40	36
LA	-1.28	-0.59	-0.18	36	46	42
MA	-0.40	1.79	2.32	10	3	4
MD	-0.50	1.60	2.27	16	5	6
ME	-0.02	1.88	2.37	2	2	3
MI	-0.91	0.98	1.36	29	16	19
MN	-0.30	1.58	2.00	8	7	12
MO	-1.48	0.24	0.52	42	31	30
MS	-1.51	-1.23	-0.38	49	51	45
MT	-0.69	0.35	0.69	21	30	28
NC	-1.17	0.75	0.98	34	22	25
ND	-1.16	-0.68	-0.68	32	47	50
NE	-1.51	-1.00	-0.60	47	50	48
NH	-0.41	0.74	1.39	11	24	17
NJ	-0.24	1.14	2.02	6	13	11
NM	-1.49	0.87	1.15	43	18	22
NV	-0.44	0.79	1.36	13	21	20
NY	-0.48	1.59	2.65	14	6	1
OH	-0.71	0.57	0.59	22	27	29
OK	-1.14	0.06	0.12	31	33	37
OR	-0.18	1.40	1.88	4	10	13
PA	-0.91	0.53	1.43	28	28	16
RI	-0.87	1.43	2.21	27	8	8
SC	-1.48	-0.08	0.26	41	37	34
SD	-1.16	-0.09	-0.09	33	38	40
TN	-1.50	-0.48	-0.50	45	44	47
TX	-0.25	0.21	0.42	7	32	31
UT	-1.51	-0.04	0.25	48	35	35
VA	-0.75	0.52	1.03	23	29	24
VT	0.09	1.29	2.31	1	11	5
WA	-0.80	1.42	2.20	25	9	9
WI	-0.37	1.12	1.15	9	14	21
WV	-1.51	-0.51	-0.72	50	45	51
WY	-1.48	-0.80	-0.68	40	49	49

Table S1: Estimates of climate policy stringency estimates and rankings (high to low)

2 Discrimination parameter estimates

Here we show the estimated discrimination parameter for each policy included in the climate policy index. The discrimination parameter is analogous to a slope, indicating the degree of change in the latent variable (climate policy) associated with the adoption of each policy.

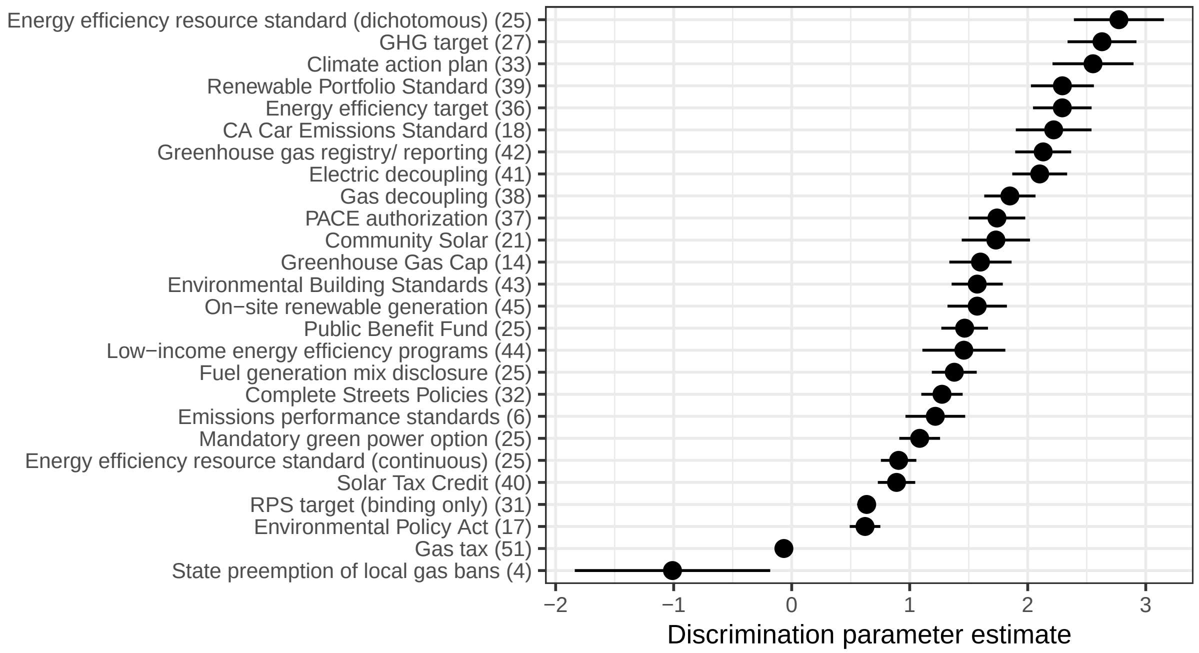

Figure S3: Discrimination parameters for the policies included in the climate policy index: The figure shows point estimates and 95% intervals defined by the 2.5th and 97.5th quantiles of the posterior distribution for each discrimination parameter estimate. Discriminaton parameters of higher magnitude indicate that the policy is strongly related to our latent climate policy index, whereas discrimination parameters with values close to zero are weakly related to the latent climate policy index. Positive discrimination parameters indicate that the adoption of the policy is associated with an increase in the latent climate policy index, whereas negative discrimination parameters indicate that the adoption of the policy is associated with a decrease in the latent climate policy index.

3 Validating the estimates of climate policy

Having illustrated the face validity of the climate policy estimates in Figures 1 and 2 from the main text, here we conduct a more systematic validation of the climate policy index. We begin with convergent validation,[1] documenting the strong cross-sectional relationships between our estimates and existing measures of state energy and climate policy. We then turn to construct validation, demonstrating that our climate policy scale is also highly correlated with measures of theoretically related concepts, such as state policy liberalism[2,3] and the ideological preferences of the mass public.[3,4]

3.1 Convergent Validation

If our estimates provide a valid measure of policy liberalism, they should be strongly related to other (valid) measures of the same concept. We assess the convergent validity of the climate policy index by comparing it with the American Council for an Energy-Efficient Economy (ACEEE) state-level energy efficiency score cards.[5] ACEEE has been ranking states on their efforts to promote energy efficiency each year since 2006.[1] Thus, a strong correlation between our climate policy index and the ACEEE scorecards provides an indication of the convergent validity of our index.

Figure S4 shows the correlation between our index and the ACEEE scorecard in 2006, 2010, 2015, and 2020. Across all years, we find that the climate policy index is strongly correlated with state scores on ACEEE's scorecard of state energy efficiency. Moreover, the correlation between our index and ACEEE's scorecard has increased overtime.

3.2 Construct Validation

The purpose of construct validation is to demonstrate that a measure conforms to well-established hypotheses relating the concept being measured to other concepts.[1] One such hypothesis is that the strength of a state's commitment to promoting a clean-energy transition should be positively correlated with its policy liberalism across the broader state policy agenda. We measure state policy liberalism based on the one-dimensional estimates from Caughey and Warshaw[2]. In Figure S5, we show that states with more liberal policies on the wider state policy agenda have more stringent climate policies.

Another such hypothesis is that the stringency of state climate policies should be correlated with the ideological preferences of the mass public. We measure ideological preferences of the mass public based on the one-dimensional estimates from Tausanovitch and Warshaw[4]. In Figure S6, we show that states with more liberal publics have more stringent climate policies.

[1]While our index does include some of the policies included in the ACEEE score cards, we include many policies not contained in the scorecards.

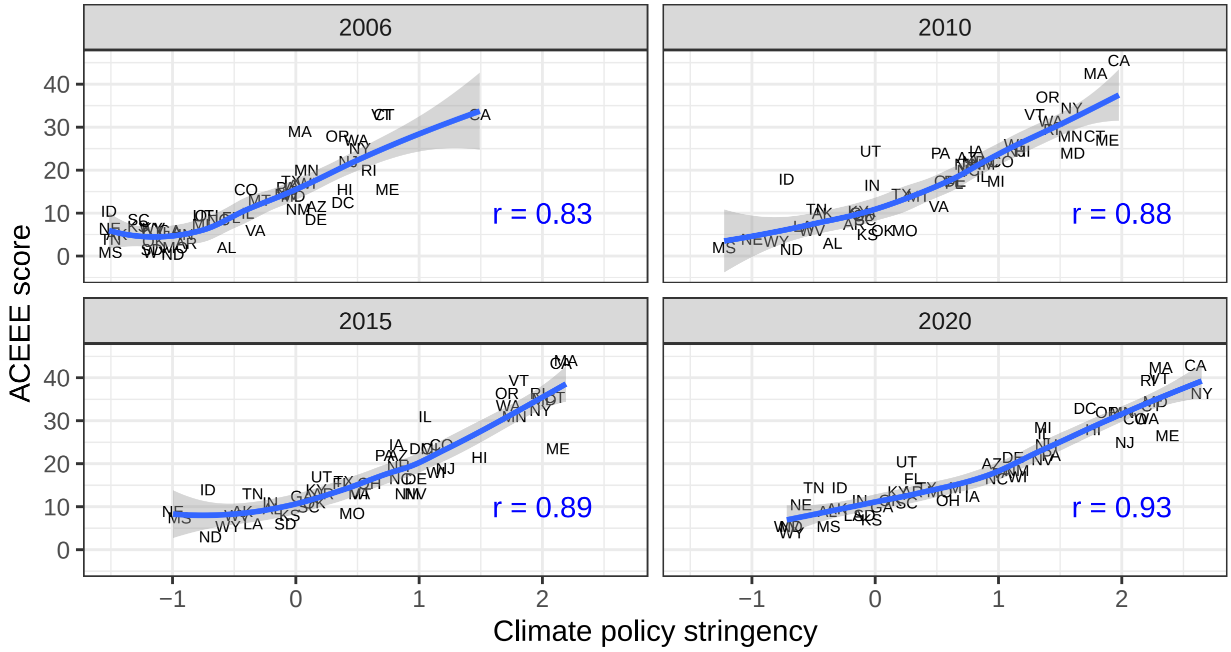

Figure S4: Validation: climate policy and ACEEE energy efficiency scores. The figure shows the correlation between our estimates of state climate policy and a subset of the American Council for an Energy Efficient Economy's annual scores. Locally weighted smoothing (lowess) lines are shown in blue, with gray bands indicating 95% confidence intervals.

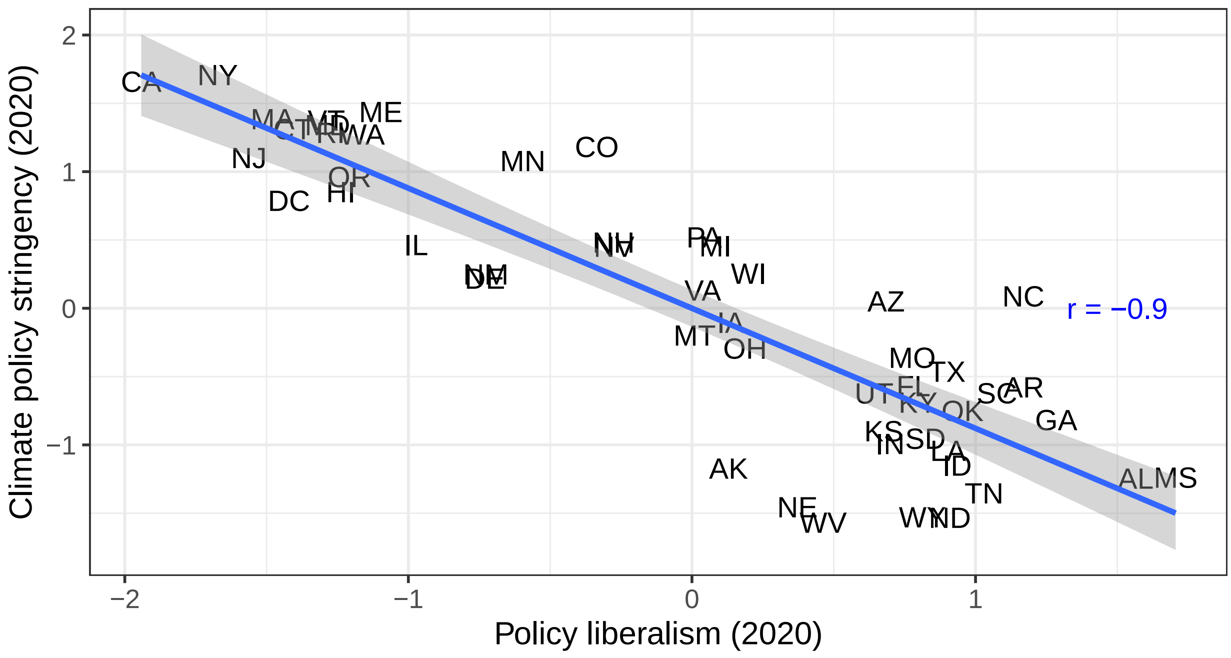

Figure S5: Construct validation: climate policy and policy liberalism. The figure shows the correlation between our estimates of climate policy and estimates of each state's policy liberalism across the entire state policy agenda. State policy liberalism is scaled such that strongly negative scores correspond to states at the far left (liberal) and strongly positive scores correspond to states on the far right (conservative). The linear best-fit line is shown in blue, with a gray band indicating the 95% confidence interval.

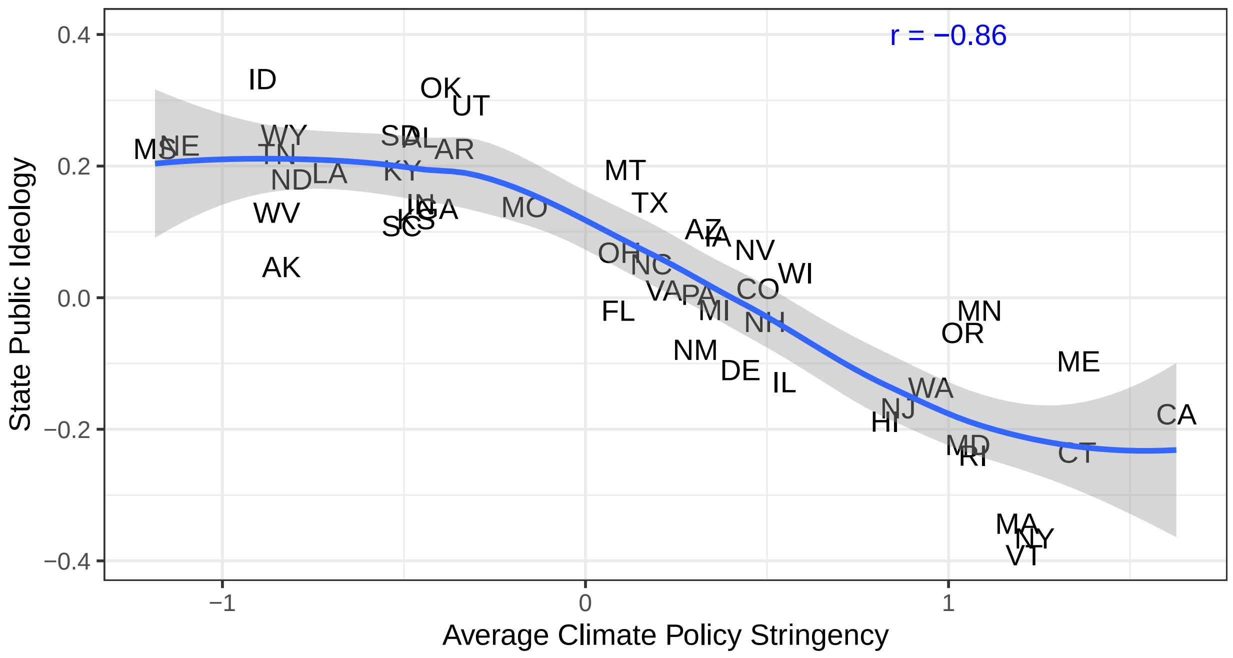

Figure S6: Construct validation: climate policy and mass ideology. The figure shows the correlation between our estimates of climate policy and estimates of the mass public's left-right ideology in each state. Public ideology is scaled such that strongly negative scores correspond to individuals at the far left and strongly positive scores correspond to individuals on the far right. The locally weighted smoothing (lowess) line is shown in blue, with a gray band indicating the 95% confidence interval around this line.

4 Regression results

	Unadjusted results			*Measurement-error corrected results*		
Dependent Variable	Estimate	Std. error	T statistic	Estimate	Std. error	T statistic
co2 (electricity) per capita	-0.113	0.037	-3.013	-0.054	0.024	-2.295
co2 (total) per capita	-0.042	0.016	-2.566	-0.020	0.010	-2.060
co2 (electricity)	-0.117	0.038	-3.045	-0.051	0.026	-2.000
co2 (total)	-0.046	0.021	-2.165	-0.021	0.011	-1.899
Coal elec. gen.	-0.055	0.071	-0.772	-0.024	0.043	-0.545
Coal energy prod.	-1.202	1.213	-0.991	-0.433	0.574	-0.754
Total energy prod.	-0.155	0.087	-1.772	-0.070	0.046	-1.526
Gas energy prod.	-0.142	0.444	-0.320	-0.119	0.261	-0.457
Ren. energy prod.	0.019	0.092	0.209	0.013	0.047	0.266
Solar elec. gen.	0.366	0.788	0.465	0.098	0.324	0.303
Gas elec. gen.	-0.239	0.221	-1.081	-0.108	0.105	-1.034
Hydro elec. gen.	0.016	0.055	0.283	0.013	0.035	0.369
Ren. elec. gen.	0.355	0.416	0.854	0.170	0.277	0.614
Total elec. prod.	-0.092	0.034	-2.722	-0.040	0.021	-1.875
Wind elec. gen.	0.456	0.649	0.703	0.157	0.361	0.436
End-use coal cons.	0.437	0.569	0.768	0.211	0.321	0.657
End-use elec. cons. (retail)	-0.078	0.029	-2.651	-0.033	0.014	-2.298
Fossil fuel cons.	0.012	0.019	0.626	0.005	0.009	0.546
End-use gas cons.	-0.033	0.020	-1.636	-0.018	0.011	-1.623
Ren. energy cons.	-0.027	0.094	-0.286	-0.007	0.046	-0.145
End-use solar energy cons.	-0.120	0.449	-0.267	-0.032	0.223	-0.146
Total elec. cons.	-0.066	0.026	-2.500	-0.029	0.014	-2.010
Total end-use elec. cons.	-0.066	0.026	-2.500	-0.028	0.013	-2.094
End-use wind energy cons.	2.400	1.234	1.946	0.992	0.624	1.589
Electricity price	0.018	0.025	0.712	0.007	0.012	0.571
GDP per capita	-0.041	0.027	-1.541	-0.015	0.012	-1.258
Wages per worker	-0.021	0.018	-1.199	-0.010	0.009	-1.136
Jobs per capita	-0.012	0.007	-1.660	-0.006	0.004	-1.420

Table S2: Regression results with and without measurement-error correction: The table shows the results from the regressions shown in Figure 5 in the main paper with and without corrections for measurement error in the climate policy index.[6] All dependent variables are logged, and the climate policy variable is scaled to have a mean of zero and standard deviation of one. Effects are estimated with OLS regression, including state and region-year fixed effects. Standard errors are clustered by state and region-year.

5 Robustness checks

Here we investigate the sensitivity of our results to the inclusion or exclusion of any of the policies included in our index. First, we estimated a series of climate policy indices, each of which excludes one of the policies included in our full index. The models are estimated in exactly the same way, except that one policy is left out each time. Next, we estimate the effect of climate policy on CO_2 emissions from the electricity sector with each index, as a robustness check to ensure that the results are not overly sensitive to the inclusion or

exclusion of any of the policies. The results are shown in Figure S7 and show that our results are robust to the specific policies included in the model. The point estimates vary slightly, but they are almost all statistically significant ($p \leq 0.05$) and similar in magnitude.

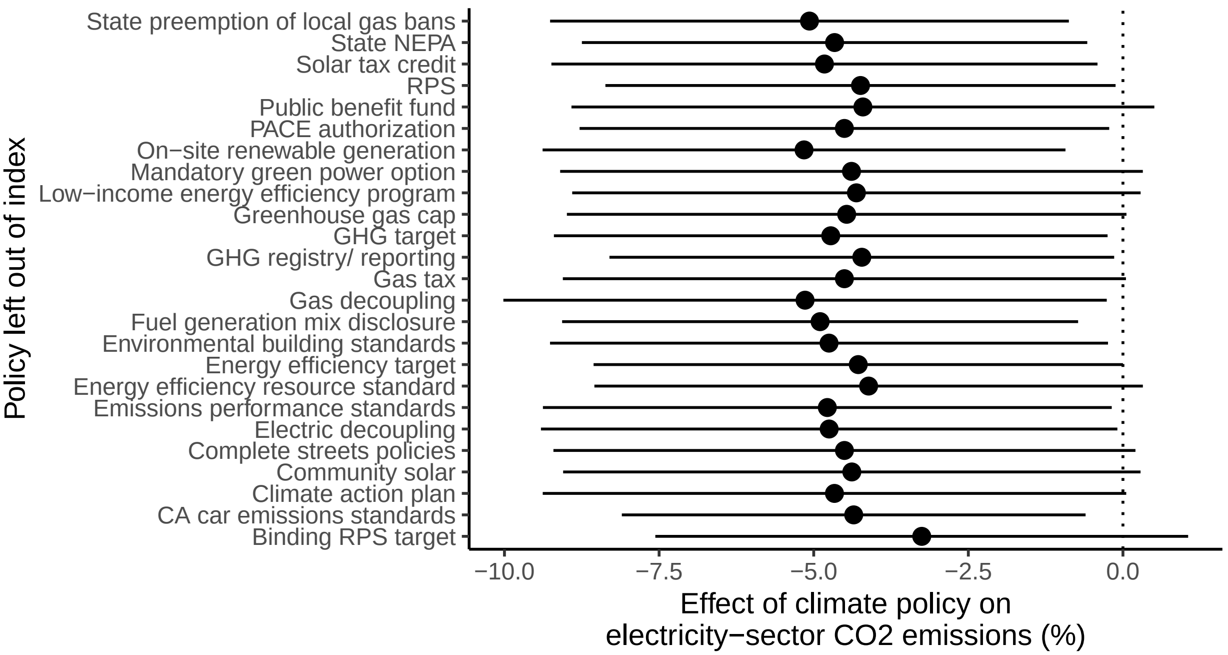

Figure S7: Sensitivity of results to the exclusion of specific policies: We estimated our climate policy index in a series of models, each of which excludes one policy that is included in the index used in our primary results. The figure shows the results of our main regression specification, with each version of the model used as the primary independent variable. The figure shows the point estimates and 95% confidence intervals from each regression (n=1,071: 51 states, observed over 21 years), accounting for measurement error in the index. The Y axis reflects the policy that was left out of the index for each set of results.

We next investigate the robustness of our results to alternative regression specifications. First, we add lagged economic indicators to our main specification, with the CO_2 emissions dependent variables. The results are shown in Figure S8. The direction, magnitude, and precision of the results are consistent with the main results presented in the paper.

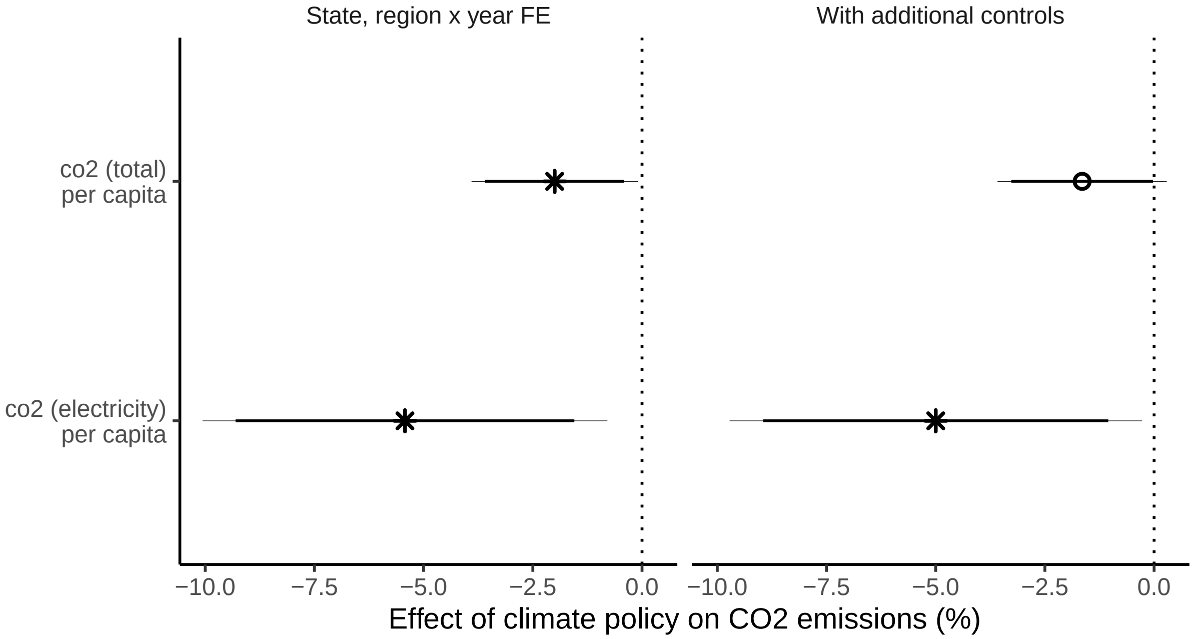

Figure S8: Results with controls for lagged economic indicators: The figure shows point estimates, 90% (thick lines) and 95% (thin lines) confidence intervals from our main regression specification including state and region-year fixed effects (left panel), and results from regression specifications including state and region-year fixed effects along with one-year lags for GDP per capita, wages per worker, and jobs per capita. In all models, n=1,071: 51 states, observed over 21 years.

Second, we run a robustness check in which we add lagged unionization rate to our models assessing the effect of climate policy on economic indicators. These results are shown in Figure S9 below. In this case, some of the coefficients are different in sign, but they remain statistically insignificant.

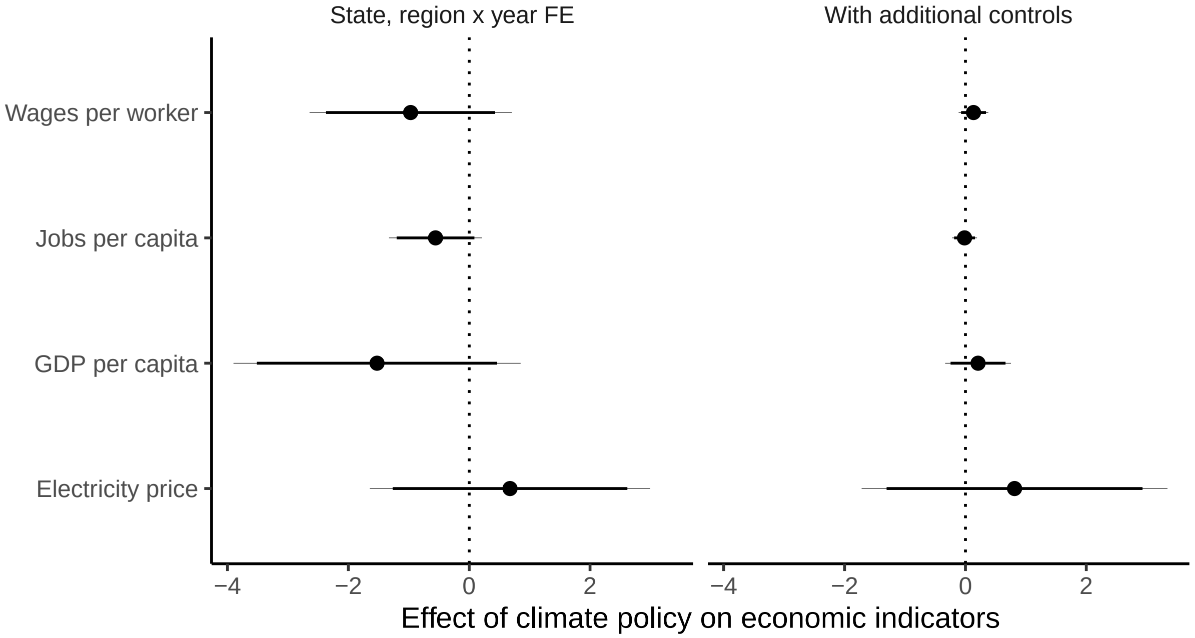

Figure S9: Results with controls for lagged unionization rate: The figure shows the results from our main regression specification including state and region-year fixed effects (left panel), and results from regression specifications including state and region-year fixed effects along with one-year lags for unionization rates in each state.Point estimates are shown with 90% (thick lines) and 95% (thin lines) confidence intervals. While the signs on some of the coefficients from these regressions vary from the main results, the pattern of statistical significance is largely consistent with the main results. In all models, n=1,071: 51 states, observed over 21 years.

6 Policy coding and data sources

Table S3: Policies included in the climate policy index

Short description	Code in policies dataset	Longer description	Data sources
Climate action plan	climate_action_plan_21	Does the state have a plan that details steps it will take to address climate change? (0 = no; 1 = yes)	Center for Climate and Energy Solutions; Morey and Kirsch (2016): Retail Choice in Electricity: What have we Learned in 20 Years?. EMRF.
Community Solar	community_solar	Does a state have a community solar program? (0=no; 1=yes)	National Renewable Energy Laboratory (NREL)
CA Car Emissions Standard	environment_CA_car_emissions_standards_21	Does the state adopt California's Car emissions standards (which are more stringent than the federal level)? (0 = no; 1 = yes)	Maryland Department of the Environment. States adopting California's clean car standards; Wikipedia "United States vehicle emission standards"
Greenhouse Gas Cap	environment_ghg_cap_21	Does the state have a binding cap on greenhouse gas emissions in the utility sector (eg; RGGI; WCI)? (0 = no; 1 = yes)	C2ES; Jonathan L. Ramseur: The regional greenhouse gas initiative: Lessons learned and issues for policy makers; California Environmental Protection Agency: Assembly Bill 32 overview.
State preemption of local gas bans	environment_preemption_naturalgasbans	Does the state prohibit local bans on gas hookups in buildings? 0=No; 1=Yes	NPR All Things Considered: "As Cities Grapple With Climate Change; Gas Utilities Fight To Stay In Business"
Public Benefit Fund	environment_publicbenefit_funds_21	Does the state have a public benefit fund for renewable energy and energy efficiency? (0 = no; 1 = yes)	C2ES; American Council for an Energy Efficient Economy (ACEEE); Database of State Incentives for Renewables and Efficiency (DSIRE); National Governors Association: Clean electricity: Public benefits funds.
Fuel generation mix disclosure	fgd_21	Does the state require electricity providers to disclose their fuel sources? (0 = no; 1 = yes)	DSIRE; Prasad; M. and Munch: S. State-level renewable electricity policies and reductions in carbon emissions. (Energy Policy; 2012)
Emissions performance standards	ghg_standards_21	Does the state have performance standards designed to reduce CO2 emissions? (0 = no; 1 = yes)	C2ES; Morey and Kirsch: Retail Choice in Electricity: What have we Learned in 20 Years?. (EMRF; 2016)
On-site renewable generation	netmeter_yearadopted_21	0 = net metering not adopted; 1 = net metering or comparable policy (feed-in tariff) adopted	Correlates of State Policy Project (CSPP)
PACE authorization	pace_21	Has the state authorized Property Assessed Clean Energy programs? (0=no; 1=yes)	PACENation; DSIRE
Environmental Building Standards	public_building_standards	Does the state have energy efficiency and other green building requirements for public buildings? (0=no; 1=yes)	CSPP
Complete Streets Policies	w_complete_streets_21	Does the state have complete streets policies? (0 = none; 1 = plan/design guide; 2 = laws/ordinances/resolutions/directives)	SmartGrowth America
Energy efficiency target	w_ee_21	Does the state have energy efficiency targets? (0 = none; 1 = voluntary/unenforceable; 2 = mandatory)	C2ES; Morey and Kirsch: Retail Choice in Electricity: What have we Learned in 20 Years?. (EMRF; 2016)

Policies included in the climate policy index S3 Continued from previous page

Short description	Code in policies dataset	Longer description	Data sources
Solar Tax Credit	w_Environment_Solar_TaxCredit_21	Does the state have a tax credit for residential solar installations? (0 = No legislation; 1 = State approved local option; 2 = State mandated credit or exemption system)	sunrun.com; energysage.com; Wholesale Solar; Lexis Nexis/State Governments: State statutes
Environmental Policy Act	w_environment_state_nepas_21	Does the state have its own version of the federal National Environmental Policy Act? (0 = none; 1 = applies to state governments only; 2 =also applies to local governments and/or private entities)	ballotpedia.org; Marchman: "Little NEPAs:" State equivalents to the National Environmental Policy act in Indiana; Minnesota; and Wisconsin (2012).Lexis Nexis/State Governments: State statutes Yost: NEPA's progeny: State environmental policy acts. (News and Analysis; 3(1) 1973)
Greenhouse gas registry/ reporting	w_gg_rr_21	Does the state require all power plants to register and record their emissions? (0 = none; 1 = voluntary; 2 = mandatory)	C2ES; Morey and Kirsch: Retail Choice in Electricity: What have we Learned in 20 Years?. (EMRF; 2016)
GHG target	w_ghg_targets_21	Does the state have a goal for emission reduction levels by a certain time period? (0 = none; 1 = executive order; 2 = statutory/public utilty commission order)	C2ES; Morey and Kirsch: Retail Choice in Electricity: What have we Learned in 20 Years?. (EMRF; 2016)
Low-income energy efficiency programs	w_low_income_ee_21	Does the state have energy efficiency programs for low-income individuals? (0 = none; 1 = legislative/regulatory requirements; utility cost-effectivenesss rules established; OR levels of spending on low-income EE less than 6.50 USD per low-income resident; 2 = legislative/regulatory requirements and utility cost-effectivenesss rules established; or levels of spending on low-income EE less than 13 USD per low-income resident)	ACEEE
Mandatory green power option	w_mgpo_21	Does the state require utilities to offer customers electricity generated from renewable sources? (0 = none; 1 = voluntary; 2 = mandatory)	C2ES; Morey and Kirsch: Retail Choice in Electricity: What have we Learned in 20 Years?. (EMRF; 2016)
Electric decoupling	w4_electric_decoupling_21	Does the state compensate utilities for selling less electricity? (0 = none; 1 = performance based incentives; 2 = non-decoupling energy efficiency adjustment; 3 = decoupled)	C2ES; Morey and Kirsch: Retail Choice in Electricity: What have we Learned in 20 Years?. (EMRF; 2016)
Renewable Portfolio Standard	w4_environment_state_rps_21	Does a state have an RPS? (0 = no target; 1 = voluntary or less than 1percent; 2 = mandatory less than 100percent; 3 = mandatory 100percent)	National Council of State Legislatures (NCSL)

Policies included in the climate policy index S3 Continued from previous page

Short description	Code in policies dataset	Longer description	Data sources
Gas decoupling	w4_gas_decoupling_21	Does the state compensate utilities for selling less gas? (0 = none; 1 = performance based incentives; 2 = non-decoupling energy efficiency adjustment; 3 = decoupled)	C2ES; ACEEE; NCSL
Energy efficiency resource standard (continuous)	x_eers	What is the state's energy efficiency resource standard (EERS)? An EERS is a quantitative; long-term energy savings target for utilities that requires them to procure a percentage of their future electricity and natural gas needs using energy efficiency measures. (continuous percent)	ACEEE
RPS target (binding only)	x_RPS_targets_bindingonly	What is the state's RPS target (enforceable standards only)? (continuous percent)	DSIRE; Solomon and Zhou (2021): Renewable Portfolio Standards: Do Voluntary Goals vs. Mandatory Standards Make a Difference? (Review of Policy Research)
Gas tax	z_gasoline_tax	What is the state's gas tax level?	Tax Foundation and American Petroleum Institute

1

7 Dependent variables and covariates: Coding and data sources

Type	Variable names	Description	Source	Filename
emissions	co2_current; so2_current; nox_current	US electric power industry estimated emissions by state (metric tons)	US Energy Information Administration (EIA): Electricity historical state data	EIA_emission_annual_1990-2020.csv
emissions	co2otaleia_current	State energy-related CO2 emissions (MMT)	EIA: Environment Data	EIA_energy.csv
mechanism	coal_current (CLPRP: LPRK to convert to BBTU); noncombustible.renew_current (NCPRB); renewableprod_current (REPRB); energyprod_current (TEPRB); gas_current (NGMPB)	Primary energy production and consumption by source; source-specific variables are percent of energyprod_current	EIA: State energy data system (SEDS)	EIA_StateEnergyProduction.xlsx
mechanism	totalgen_current; coalgen_current; gasgen_current; oilgen_current; windgen_current; solargen_current	Net electricity generation by type of producer by energy source; filtered to include total electric power industry as producer; source-specific variables are percent of totalgen_current	EIA: Electricity historical state data	EIA_annual_generation_state.csv
mechanism	TETCB	Total energy consumption in billion BTU	EIA: SEDS	EIA_annual_totalconsumption_state.csv
outcome	electricityprice_current	Average electricity price in cents per kwh	EIA: Electricity data browser	EIA_electricity_price.csv
outcome and covariate	wages_current	Wages and salaries (USD1000s)	US Bureau of Economic Analysis (BEA)	SAINC6N__abb_1998_2020.csv
outcome and covariate	gdp_current	State GDP (million USD)	BEA	SAGDP2N_abb_1997_2021.csv
outcome and covariate	jobs_current	Total employment (number of jobs) aggregated across NAICS codes	BEA	
population	pop	State population totals	US Census Bureau	various
covariate	unionizationrate	Percent of workers who are members of labor unions	Hirsch and Macpherson "Union Membership and coverage database from the current population survey: Note" (2003) (unionstats.com)	union_membership.csv

Table S4: **Sources and descriptions of outcome and covariate data**

Supplementary Information References

1. Robert Adcock and David Collier. Measurement Validity: A Shared Standard for Qualitative and Quantitative Research. *American Political Science Review*, 95(3):529–546, 2001.

2. Devin Caughey and Christopher Warshaw. The dynamics of state policy liberalism, 1936–2014. *American Journal of Political Science*, 60(4):899–913, 2016.

3. Devin Caughey and Christopher Warshaw. Policy Preferences and Policy Change: Dynamic Responsiveness in the American States, 1936–2014. *American Political Science Review*, 112(2):249–266, May 2018.

4. Chris Tausanovitch and Christopher Warshaw. Measuring Constituent Policy Preferences in Congress, State Legislatures and Cities. *Journal of Politics*, 75(2):330–342, 2013.

5. ACEEE. The state energy efficiency scorecard, april 2022. URL `https://www.aceee.org/state-policy/scorecard`.

6. Shawn Treier and Simon Jackman. Democracy as a latent variable. *American Journal of Political Science*, 52(1):201–217, 2008.
