# Supplementary material for: How climate policy commitments influence energy systems and the economies of US states
Source: Nat Commun. 2023 Aug 10;14:4850. doi: 10.1038/s41467-023-40560-y (PMC10415253; doi:10.1038/s41467-023-40560-y)
Supplement: Supplementary file 3 — Reporting Summary [file 41467_2023_40560_MOESM3_ESM.pdf]

nature portfolio

Corresponding author(s): Parrish Bergquist

Last updated by author(s): June 14, 2023

Reporting Summary

Nature Portfolio wishes to improve the reproducibility of the work that we publish. This form provides structure for consistency and transparency in reporting. For further information on Nature Portfolio policies, see our Editorial Policies and the Editorial Policy Checklist.

Statistics

For all statistical analyses, confirm that the following items are present in the figure legend, table legend, main text, or Methods section.

n/a	Confirmed	
☐	☒	The exact sample size (*n*) for each experimental group/condition, given as a discrete number and unit of measurement
☐	☒	A statement on whether measurements were taken from distinct samples or whether the same sample was measured repeatedly
☐	☒	The statistical test(s) used AND whether they are one- or two-sided<br>*Only common tests should be described solely by name; describe more complex techniques in the Methods section.*
☐	☒	A description of all covariates tested
☐	☒	A description of any assumptions or corrections, such as tests of normality and adjustment for multiple comparisons
☐	☒	A full description of the statistical parameters including central tendency (e.g. means) or other basic estimates (e.g. regression coefficient) AND variation (e.g. standard deviation) or associated estimates of uncertainty (e.g. confidence intervals)
☐	☒	For null hypothesis testing, the test statistic (e.g. *F*, *t*, *r*) with confidence intervals, effect sizes, degrees of freedom and *P* value noted<br>*Give P values as exact values whenever suitable.*
☐	☒	For Bayesian analysis, information on the choice of priors and Markov chain Monte Carlo settings
☐	☒	For hierarchical and complex designs, identification of the appropriate level for tests and full reporting of outcomes
☐	☒	Estimates of effect sizes (e.g. Cohen's *d*, Pearson's *r*), indicating how they were calculated

Our web collection on statistics for biologists contains articles on many of the points above.

Software and code

Policy information about availability of computer code

Data collection	No specialized software was used for data collection.
Data analysis	We used the R package dbmm to estimate our climate policy index, with version 0.5.3 of cmdstanr, which is the interface that dbmm uses to operate Stan within R. We have included a citation to the dbmm package in the methods section. The code used to estimate the model has been posted to the Harvard Dataverse, along with the code used to produce all tables and figures in the main text and supplementary information.

For manuscripts utilizing custom algorithms or software that are central to the research but not yet described in published literature, software must be made available to editors and reviewers. We strongly encourage code deposition in a community repository (e.g. GitHub). See the Nature Portfolio guidelines for submitting code & software for further information.

Data

Policy information about availability of data

All manuscripts must include a data availability statement. This statement should provide the following information, where applicable:

- Accession codes, unique identifiers, or web links for publicly available datasets
- A description of any restrictions on data availability
- For clinical datasets or third party data, please ensure that the statement adheres to our policy

Our data and code have been published to the Harvard Dataverse, at the link included in our data and code availability statements. The link is: https://doi.org/10.7910/DVN/PXWXWI

Human research participants

Policy information about studies involving human research participants and Sex and Gender in Research.

Reporting on sex and gender	N/A
Population characteristics	N/A
Recruitment	N/A
Ethics oversight	N/A

Note that full information on the approval of the study protocol must also be provided in the manuscript.

Field-specific reporting

Please select the one below that is the best fit for your research. If you are not sure, read the appropriate sections before making your selection.

☐ Life sciences ☒ Behavioural & social sciences ☐ Ecological, evolutionary & environmental sciences

For a reference copy of the document with all sections, see nature.com/documents/nr-reporting-summary-flat.pdf

Behavioural & social sciences study design

All studies must disclose on these points even when the disclosure is negative.

Study description	Analysis of climate policy stringency and climate policy effects for all 50 US states, from 2000-2020. Data are state-year indicators for 26 different policies, which we aggregate into a state-year index of climate policy stringency. We use this index to estimate the effects of climate policy on various indicators of carbon pollution, energy production and consumption, and economic performance. All data points are aggregated at the state-year level. No human subjects are involved in this research.
Research sample	US state climate policies, 2000-2020. Our sample includes an annual measure of climate policy stringency and an array of outcome variables described in the study, for each state between these years. This time period was chosen because it is the time period during which states have been enacting climate policy. All US states are included. These units thus represent a census of the population of US states enacting climate policies.
Sampling strategy	The sample of states represents a census of the population of US states during the time period in which states have been active in enacting climate policies.
Data collection	We compile the policy data from advocacy groups, government websites, and academic sources. We began by reviewing published work on this topic and gathering time-series data on every policy that had been included in prior analyses of state climate policies or state policy more broadly. Next, we consulted the websites of several prominent NGOs that aggregate data on state climate policy. From these websites we downloaded the policy data or, if the policy data were not available from the NGO, we obtained it from state websites. Next, we supplemented this data with policies that we knew to exist due to our domain area expertise in climate and energy policy. Data were recorded digitally using spreadsheets.
Timing	data were collected between January 2019 and November 2022
Data exclusions	No data were excluded
Non-participation	No participants were involved.

Randomization	This study is not a randomized control trial.

Reporting for specific materials, systems and methods

We require information from authors about some types of materials, experimental systems and methods used in many studies. Here, indicate whether each material, system or method listed is relevant to your study. If you are not sure if a list item applies to your research, read the appropriate section before selecting a response.

Materials & experimental systems

n/a	Involved in the study
☒	☐ Antibodies
☒	☐ Eukaryotic cell lines
☒	☐ Palaeontology and archaeology
☒	☐ Animals and other organisms
☒	☐ Clinical data
☒	☐ Dual use research of concern

Methods

n/a	Involved in the study
☒	☐ ChIP-seq
☒	☐ Flow cytometry
☒	☐ MRI-based neuroimaging
